# The health needs and access barriers among refugees and asylum-seekers in Malaysia: a qualitative study

**DOI:** 10.1186/s12939-018-0833-x

**Published:** 2018-08-15

**Authors:** Fiona Leh Hoon Chuah, Sok Teng Tan, Jason Yeo, Helena Legido-Quigley

**Affiliations:** 1 Saw Swee Hock School of Public Health, National University of Singapore and National University Health System, Singapore, Singapore; 2 United Nations High Commissioner for Refugees, Kuala Lumpur, Malaysia; 3 Universiti Putra Malaysia, Serdang, Selangor Malaysia; 4 London School of Hygiene and Tropical Medicine, London, UK

**Keywords:** Malaysia, Refugees, Asylum-seekers, Urban refugees, Healthcare access, Health needs, Health system, Forced migration

## Abstract

**Background:**

In Malaysia, refugees and asylum-seekers are a vulnerable group that often face circumstances in which their health and wellbeing can be compromised. This qualitative study sought to examine the key health concerns and barriers to healthcare access among refugees and asylum-seekers in Malaysia through the lens of healthcare professionals, program staff and experts on refugee and migrant health.

**Methods:**

We conducted 20 semi-structured in-depth interviews with experts, healthcare professionals, program managers or executives from UN agencies, public healthcare facilities, civil society organizations, and academic institutions in Malaysia. Interviews were transcribed and analyzed both deductively and inductively using thematic analysis.

**Results:**

Participant narratives highlight that the health needs of refugees and asylum-seekers in Malaysia are complex. As reported, access to healthcare is underpinned by numerous social, cultural and economic determinants compounded by a legal environment that lacks inclusivity of refugees and asylum-seekers. Apart from the health risks associated with the migration process, limited access to comprehensive healthcare post-arrival remain a problem for refugees and asylum-seekers in Malaysia. Key barriers to healthcare access are linked to poor health literacy and the lack of awareness on one’s right to healthcare; language and cultural differences; protection issues resulting from a lack of legal status; and an inability to afford healthcare due to inadequate livelihoods. Overall, poor access to healthcare is perceived to have detrimental consequences on the health status of refugees, asylum-seekers and its host population, and may incur greater costs to the health system in the long run.

**Conclusion:**

Comprehensive efforts in practice and research that tackle the social, cultural and economic determinants of health, and more inclusive health policies are crucial in strengthening healthcare access among refugees and asylum-seekers in Malaysia. Practical recommendations include improving the health literacy of refugees and asylum-seekers for better navigation of the health system; bridging language and cultural gaps through translation support and inter-cultural orientation; implementing policies grounded in the right to healthcare for all regardless of legal status and in the interest of public health; and establishing a larger evidence base to drive policy development and implementation for refugee health within the Malaysian context.

## Background

Access to health care is fundamental in attaining good health and it encompasses several dimensions based on one’s capitals and how a health system is organized [1]. Refugees and asylum-seekers often encounter circumstances in which their health and well-being are compromised. Despite these health needs, access to health care for refugees is often restricted in host countries, and this is exacerbated by various reasons such as a lack of inclusive policies, language and cultural barriers, financial ability to afford, and legal status [2].

Apart from the health risks associated with the dangerous land and sea voyages that most undertake to flee persecution [3, 4], refugees and asylum-seekers frequently wind up in protracted situations of overcrowding [5, 6], food insecurity [7, 8], incomplete water and sanitation provisions [5, 7, 9], and poverty in the absence of adequate livelihoods [10, 11]. In Malaysia, refugees and asylum-seekers live in urban settings instead of camps [12]. The urban environment coupled with population ageing due to protracted displacement, presents complex healthcare needs among the refugee and asylum-seeker population [13]. Further, as Malaysia is not a state party of the 1951 Convention Relating to the Status of Refugees and its’ 1967 Protocol [14], efforts to address these needs are complicated by numerous legal and political challenges.

In Malaysia, there are some 157,580 refugees and asylum-seekers registered with the United Nations High Commissioner for Refugees (UNHCR) [15]. Due to a policy environment that lacks inclusivity of undocumented migrants; refugees and asylum-seekers in Malaysia do not have access to basic services including legal employment, formal education and free healthcare [9]. Despite this, UNHCR in Malaysia had successfully negotiated a Memorandum of Understanding with the Ministry of Health (MoH) in 2005 for refugees to be accorded a 50% discount off the foreigners’ rate at public healthcare facilities [11, 16]. In theory, refugees could receive health services at public health facilities; however, the cost of medical care after discount remains exorbitant and always incurs high out-of-pocket payments, thus effectively compromises their ability to seek healthcare services [9, 11]. To reduce such barriers and improve access to secondary and tertiary medical care, UNHCR initiated a health insurance program in 2014 named REMEDI for refugees in collaboration with a private insurance company [17, 18]. Nonetheless, inadequate access to healthcare among refugees and asylum-seekers remains a concern given the myriad of barriers in the form of security and protection issues due to a lack of legal status; language and cultural differences with the host population; and poor health literacy [19].

In 2015, UNHCR conducted a health access and utilization survey (HAUS) among 591 urban refugees and asylum-seekers living throughout Malaysia [20]. Based on the survey results, 45.2% of those who sought healthcare went to government health facilities, 43.3% went to private facilities, and the remaining 11.5% went to facilities run by non-governmental organizations (NGOs). Overall, 26.7% of those with chronic conditions – including hypertension, asthma or chronic obstructive pulmonary disease, and Type 2 diabetes mellitus - were not able to seek treatment for their conditions. Additionally, despite the fact that 91.8% of pregnant women had at least one antenatal visit during their pregnancy, 44.6% of them reported difficulties in accessing antenatal services. In both examples, access to healthcare was challenged by an inability to afford medical care and transport costs, as well as language and communication difficulties [20]. Evidently, addressing the complex health needs and access barriers of the refugee and asylum-seeker population brings about significant challenges and resource demands, which warrants exploration in order to identify better solutions.

However, one of the major challenges in identifying potential solutions is in the dearth of academic and policy research examining the nexus between forced migration and health particularly within the Malaysian context. The lack of literature has prompted an imperative to conduct this qualitative study which aims to examine the key health concerns and barriers to healthcare access among the refugee and asylum-seeker population in Malaysia through the lens of healthcare professionals, program managers or executives, and experts on refugee and migrant health.

## Methods

This qualitative study was carried out from July 2016 to November 2017 as part of a larger research project exploring refugee health issues and responses in Southeast Asia. To address the aims of this study, the researchers sought to explore the participants’ perceptions on healthcare access among refugees and asylum-seekers in Malaysia. In a companion paper of this research project; the health responses, challenges and ways forward in addressing the health needs of refugees and asylum-seekers in Malaysia from a health systems and policy perspective are examined and reported.

### Participants and sampling

Purposive sampling was conducted to recruit key informants with professional expertise and experience working in the field of refugee health or in providing healthcare services to refugees and asylum-seekers. Potential participants were identified through a stakeholder analysis process which involved searching through organizational websites of individuals within organizations that serve refugees and migrants in Malaysia, as well as through the authors’ past professional contacts. The authors sought to include a representative sample of key informants from different organization types, i.e. UN organizations, public healthcare facilities, international civil society organizations, local civil society organizations and academia. The authors also purposively ensured that the sample included professionals from different levels, i.e. program managers, program executives, policy-makers, clinicians and academicians. Potential participants were sent an email inviting them to participate in the study along with a participant information sheet and consent form. Among those invited, 4 did not respond to the invitation and 2 refused to be interviewed due to political sensitivity, and concerns on the value of qualitative research evidence given the anonymity of participants. Following the first round of interviews, an additional 10 participants were recruited via snowball sampling techniques based on nominations by the initial 10 participants. Recruitment of participants ceased following thematic saturation, i.e. when the researchers agreed collectively that further interviews would not likely lead to new ideas or information. In total, 20 participants were interviewed. Of these, 12 were based in Malaysia, 3 held regional positions with oversight of programmatic and research work relating to refugees in Malaysia, and 5 others were refugee health experts from neighboring countries in the ASEAN region who could provide insights on the position of Malaysia in dealing with refugees. The sample characteristics are presented in Table 1.

**Table 1:** Sample characteristics

*Organization Type*	*N*
UN organizations	5
Public healthcare facilities	2
International civil society organizations	6
Local civil society organizations	4
Academia	3
*Total*	*20*
*Professional Role in relation to Refugee Work*	*N*
Program manager	7
Program executive	2
Policy and programmatic work	3
Healthcare professional	5
Academician	3
*Total*	*20*
*Background*	
Clinician	11
Allied health (e.g. pharmacy, psychology, community health)	4
Non-health (e.g. law, economics, operations)	5
*Total*	*20*

### Ethics

The research project for this study received ethical approval from the National University of Singapore Institutional Review Board (NUS-IRB). At point of recruitment, each participant was provided an information sheet with details of the study. Signed consent was obtained for participation in the study, permission to be audio-recorded, and permission to be quoted anonymously in research outputs. Participants were allowed to refuse any of these options, any questions posed to them and/or withdraw from the study at any point. The audio-recordings were anonymized prior to transcription to protect the participants’ identities. The participants’ names and identifying data were also removed from all research documents to ensure confidentiality. Given the sensitivity of the topic, a key challenge of the research was in ensuring that the findings presented would not pose any risk in causing harm to the situation for refugees and asylum-seekers in Malaysia. The researchers strived to ensure this by seeking feedback and advice from participants and other experts in the topic area on the best approach to adopt in presenting the findings of this study.

### Data collection

Two of the researchers (FLHC, HLQ) conducted in-depth interviews in English either face-to-face or via Skype with the participants, each interview lasting an average of 60 min in length. These were done at a time and site based on the participants’ availability and preference. The researchers used a semi-structured topic guide to explore the key health concerns and barriers to healthcare access among the refugee and asylum-seeker population in Malaysia. The line of questioning followed each participant’s agenda and responses as much as possible, while remaining relevant to the aims of the study. The audio recordings of the interviews were transcribed in verbatim. The field notes for one of the interviews in which the participant preferred not to be recorded were typed out into a separate note sheet.

### Data analysis

To fully immerse in the data, the authors listened to the audio recordings and read through the transcripts to correct any errors in the initial transcribing. Transcripts and field notes were then coded line-by-line using an interpretive approach which focuses on the participants’ interpretations, perceptions and the ways in which they make sense of the topic of discussion. In this process, the authors assigned a code word or phrase that most accurately described the meaning of the text segment. Once all interviews were coded, the authors organized these codes into broader themes based on categories identified from Levesque’s et al. conceptual framework on access to patient-centered healthcare in mind [1]. According to Levesque et al., the conceptualization of ‘healthcare access’ entails five key dimensions that defined our categories for identifying themes, namely: 1) Approachability; 2) Acceptability; 3) Availability and Accommodation; 4) Affordability; and 5) Appropriateness. Parallel to these dimensions are the corresponding abilities of the patients: 1) Ability to perceive; 2) Ability to seek; 3) Ability to reach; 4) Ability to pay; and 5) Ability to engage. [1] After codes were categorised within these dimensions, they were collapsed into themes. Apart from identifying themes that relate to these dimensions and the corresponding abilities, separate themes relating to the process of obtaining care and its consequences as presented in the framework were also identified. QSR NVIVO11 was used during data analysis to store and manage the data. The researchers engaged in an iterative process of developing and reviewing the themes. Regular discussions between the researchers were carried out to build consensus on the final list of themes and the main findings of each theme. Deviant cases were sought out and examined in the analysis. These included individuals who presented opinions or ideas that were different from the majority of the participants on certain topic areas. To enhance the credibility of the study findings, all participants were contacted for a member check in the final stage of preparing the manuscript to validate the researchers’ interpretation of the findings and to ensure accurate representation of the participants’ perspectives.

### Reflexivity

All researchers in this study have prior professional experiences working on refugee and/or migrant health issues, three of whom (FLHC, STT, JY) worked on health and social programs for refugees in Malaysia. By sharing relatable professional experiences with the participants; FLHC, STT and JY hold an ‘insider’ position in this study. Hence, to avoid the influence of any pre-conceived assumptions, subjectivities and prejudices; self-reflexivity was a particularly important component throughout the research process. It was also valuable to have the fourth researcher (HLQ) whose experience is more relevant to the European context, to be involved in data collection and analysis. This ensured that all topic areas in the interviews were covered comprehensively and that neutrality was maintained throughout the data collection and analysis process.

## Results

**Fig. 1:**
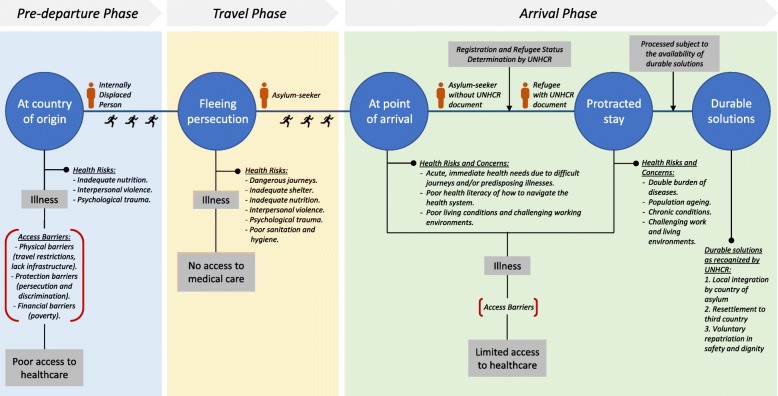
Health risks and concerns, and healthcare access among refugees and asylum-seekers along the migration journey

**Fig. 2:**
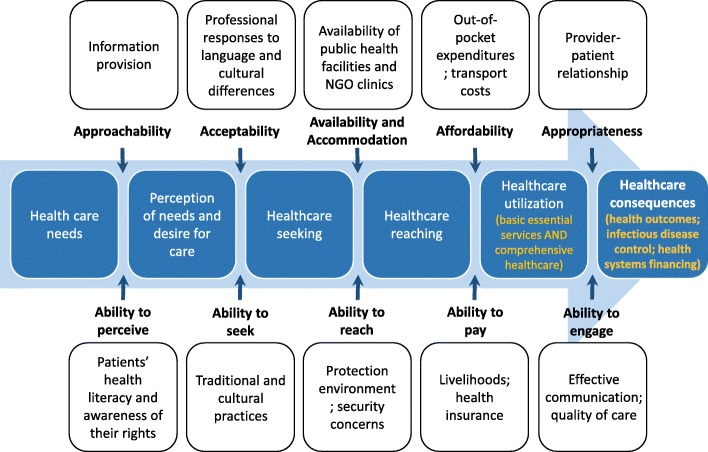
Conceptualization of healthcare access among refugees and asylum-seekers in Malaysia, as adapted from Levesque et al.’s access framework [17]

**Fig. 3:**
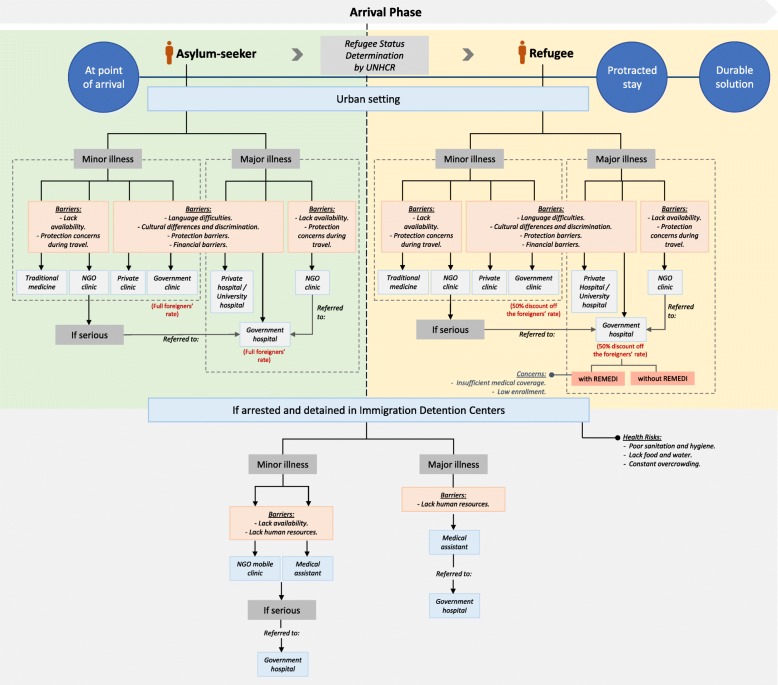
Patient pathways for healthcare seeking and access barriers faced by refugees and asylum-seekers in Malaysia

In addressing the health concerns of refugees and asylum-seekers in Malaysia, refugees’ health care needs, access to healthcare and its barriers were explored in depth. The findings are organized according to seven key themes. The first theme demonstrates the health needs of the refugees at different migration phases from the participants’ perspectives. Figure 1 represents an illustration of the key health concerns that refugees and asylum-seekers face throughout the migration journey and their ability to access healthcare. The second to sixth themes reflect Levesque’s et al. five key access dimensions and their corresponding patient abilities [1] as identified through the lens of healthcare professionals, program staff and experts in refugee and migrant health. The seventh theme was induced from the participants’ narratives highlighting the negative implications of poor healthcare access for the refugee and asylum-seeker population in Malaysia. Based on Levesque et al.’s access framework, the key elements for access among refugees and asylum-seekers in Malaysia as per this study’s findings are illustrated in Fig. 2. A summary of the key themes and its respective findings are also presented in Table 2. The patient pathways for healthcare seeking and the access barriers faced by refugees and asylum-seekers upon arriving in Malaysia is illustrated in Fig. 3.

**Table 2:** Key themes, main findings and example quotes

Key Themes	Main Findings as Reported by Participants	Quotes
Refugee’s health needs at different migration phases	• At the pre-departure phase, health problems are precipitated by exposure to interpersonal violence, inadequate nutrition, psychological trauma due to hardship, and poor access to healthcare. Healthcare access at this phase is undermined by travel restrictions, lack of infrastructure, persecution and discrimination. • During travelling phase, health problems are precipitated by inadequate food and nutrition, sub-standard shelter, poor sanitation and hygiene, interpersonal violence, and psychological trauma. Medical care during their journey is usually non-existent. • Health risks associated with difficult journey and poor health conditions at country of origin often manifest upon arrival at host countries. • Refugee’s morbidities in Malaysia include communicable diseases, maternal and child health related conditions, malnutrition, psychosocial or mental health disorders, and non-communicable diseases.	*“I think it’s throughout the whole journey itself. When they leave the country, back home their condition might not be ideal, so there’s already some history behind it. During the movement, it’s difficult to maintain your hygiene and health. And then when you arrive, it’s a totally new environment.” (I07)* *“The conditions on-board were pretty terrible. […] People were packed, couldn’t move, weren’t allowed to move, fed very little – usually just a bowl of rice, a cup of water a day. Sanitary conditions were kind of non-existent […] people were being killed on-board, probably as a way to keep discipline on-board so the people who tried to kind of start trouble or ask for more food or ask to move around would be beaten and sometimes fatally.” (I17)*
‘Approachability and Ability to Perceive’: Poor health literacy and the lack of awareness on one’s right to healthcare	• Refugees and asylum-seekers lack information on available health services and face difficulties navigating the health system. • Refugees and asylum-seekers lack awareness of their right to seek healthcare. • Refugees and asylum-seekers lack understanding of the treatment and administrative procedures due to language barriers which raises some ethical concerns. • Refugees and asylum-seekers are highly dependent on their community members as information sources in healthcare seeking. • NGO clinics play an important role in facilitating the process of navigating the health system through their referral procedures.	*“Sometimes, the refugee may not know they actually have the right to access the health facility, so they don’t seek help [...] it is also due to lack of information provided.” (I07)* *“There’s a lot of problem with language as well [...] most of the time, even if they signed some document, they do not know what they are signing […] without a proper interpretation or translation available.” (I01)*
‘Acceptability and Ability to Seek’: Language difficulties and cultural differences as barriers to access	• Language and communication difficulties are key barriers to access due to the refugee and asylum-seeker’s inability to speak local languages and the lack of translation services. • Public healthcare services are not tailored to the refugee and asylum-seeker’s culture and traditions. Alternative medicines like traditional medicines based on cultural practices are unavailable. • During the health seeking process, refugees and asylum-seekers may face prejudices due to differences in their nationality and culture.	*“A lot of times, even history taking is a problem. […] They probably just say, ‘Pain’ but when you want to get further history, past medical history or family history, it’s very difficult. [...] Language itself is a big problem.” (I01)* *“It’s actually on linguistic and cultural issue […] the staff in that particular facility may have difficulty to cope because they will need more time to use interpreter and more time to adjust on the culture and understanding of each other and belief.” (I07)*
‘Availability and Ability to Reach’: Protection barriers to access; and poor healthcare access in immigration detention centers	• Availability of public health facilities is not an issue as Malaysia is a country with one of the highest numbers of healthcare facilities. • The Malaysian health system should have the capacity to cover the refugee and asylum-seeker population due to its small number relative to the host population. • Refugees are provided reasonable healthcare treatment despite the legal circumstances for the population in Malaysia. • Protection barriers are linked to risks of arrest and raids, a lack of documentation, and the set-up of immigration counters at health facilities. • Availability of NGO-run clinics is limited but these clinics are easy to reach for refugees and asylum-seekers. • Conditions in IDCs are dire due to the lack of food, water, sanitation and overcrowding. Access to services is poor.	*“All in all, the capacity is there and then the quality and quantity of clinics are also there. So, I don’t see an issue about supply and demand but I see an issue of poor accessibility due to the high cost.” (I01)* *“You can understand why undocumented migrants won’t go to these clinics because they are worried about being nabbed.” (I12)* *“Scabies is now very common there because of the lack of hygiene, and everybody’s coughing and runny nose […] some get injuries […] they have gastric pain, they cannot sleep, they have headaches…the headache is very common among them partly because of no ventilation. The windows are all up-- there’s no window, actually; there’s a gap with some wire netting there, for the air to flow on the top. There’s no window. They can’t look out and when the door is shut, they don’t get to see anything […] the bathrooms are at the end, although the bathrooms are often very dirty bathrooms.” (I06)*
‘Affordability and Ability to Pay’: Financial difficulties as a key barrier to access	• Financial challenges are key barriers to healthcare access, primarily due to lack of livelihoods, no healthcare insurance, increased foreigner charges, and no UNHCR documents. • Travelling to hospital incurs high transportation costs especially for chronic patients. • Health is not a main priority for refugees because they struggle to meet other basic needs such as shelter and food. • REMEDI – the health insurance program has great potential in mitigating financial barriers to healthcare but is limited in its coverage and its enrollment is low.	*“For accessibility itself just use the cost* per se *is really very limited because of the very high cost for migrants. Availability is there but the affordability is not there. So, I will say the accessibility is really not there for the migrants.” (I01)* *“Like delivery, you know, which they used to be able to afford. But it’s gone up how many times now? From 400 ringgit, after 50% discount, now it’s gone up to, like, what, 2500 after discount? So it’s very unaffordable for many of them, and especially for those without documents […] who are not registered yet, they charge the full price. So it’s really prevented a lot of people from accessing healthcare.” (I09)* *“Yes, we have the insurance, but it’s still a challenge […] it will still take some time before people buy on to that idea.” (I09)*
‘Appropriateness and Ability to Engage’: Need for access beyond basic essential services; and lack of engagement due to communication difficulties	• Comprehensive health needs are still existent as the refugee and asylum-seeker population is here for a protracted period. • Access to healthcare should be extended to other areas including antenatal care, health education, preventive care, family planning etc. • Language difficulties serve as key barriers in enabling refugees and asylum-seekers to engage meaningfully in the healthcare seeking process.	*“[…] comprehensive health needs are still existent, being that the population is going to be here for a protracted period of time, we can’t just focus on just access to just basic services; we also need to look at the more comprehensive needs for the long-term, which will then include all the other areas.” (I09)* *“I do have patients or refugees in my clinic telling me that sometimes they don’t treat me like a human because they just poke and take blood” or “They will discuss amongst themselves and give me some medicine to take.” (I01)*
Negative implications of poor healthcare access	• Negative implications include poor health outcomes among the population, increase in home deliveries, spread of infectious diseases, poorer control of non-communicable diseases leading to secondary complications, loss to follow-up and poorer adherence rates among chronic care patients, and poorer quality of life and psychological health. • Poor access due to the MoH’s cost-cutting measures could also lead to greater financial cost to the health system in the long term.	*“I think we’ve not seen the heights of the consequences of this yet, because we don’t know how many more TB cases may not be getting access to care […] It will eventually encourage people to have home deliveries, which is not a great thing […] it’s a little bit worrisome.” (I09)* *“I guess that we are a bit short-sighted to cover for our financial deficit. We cut down unnecessary for all these important vital components which I think, in the long-term, will impact us quite badly […] the long-term impact is quite bad with increase in cost.” (I01)*

### Refugee’s health care needs at different migration phases

Overall, participants perceived that the health concerns of refugees and asylum-seekers are more complex than those of the general population. As iterated, refugees and asylum-seekers encounter a myriad of health risks throughout their migration journey. At the pre-departure phase, the health problems are precipitated by exposure to interpersonal violence, inadequate nutrition, psychological trauma due to hardship, and poor access to healthcare. At this phase, physical barriers to access are prominent due to travel restrictions, and difficulties traveling due to a lack of infrastructure and means to travel. Protection barriers also inhibit one’s ability to seek healthcare due to the persecution and discrimination they face in their home country. Financial barriers to healthcare access are also common as a result of poverty and persecution. Participants’ accounts illustrated that during the traveling phase, displaced persons often undertake precarious and dangerous journeys to flee persecution. Apart from the difficult journeys, health problems are precipitated by inadequate food and nutrition, inadequate shelter, poor sanitation and hygiene, interpersonal violence, and psychological trauma. They also have no access to medical care during their travel which leads to high morbidity and mortality.

Due to the difficult journeys during travel and poor health conditions at country of origin, participants emphasized that refugees and asylum-seekers may have acute and immediate health needs upon arriving at their country of destination such as Malaysia. As described by the participants, the risk factors that displaced persons encounter during the pre-departure and traveling phases can lead to unfavorable health outcomes post-arrival. Collectively, narratives highlight that the most concerning morbidities associated with the refugee and asylum-seeker population in Malaysia include communicable diseases particularly TB and HIV; maternal and child health related conditions; malnutrition; psychosocial or mental health disorders; and non-communicable diseases including diabetes, hypertension, cardiovascular disease and end-stage renal disease.

### ‘Approachability and Ability to Perceive’: Poor health literacy and the lack of awareness on one’s right to healthcare

This theme relates to the refugee or asylum-seeker patient’s ability to identify healthcare services and the information sources that influence one’s decisions in healthcare seeking [1]. Some participants described that refugees and asylum-seekers are rarely provided sufficient information on the services available and thus face difficulties navigating the health system upon arrival. One participant emphasized the importance of such health literacy for better access:

*“I think health information should be disseminated and health promotion should be provided to the refugee population when they actually arrive, because that sort of [helps] their understanding and also expectations […] of what they can do themselves and what they can access, what is available, and also what kind of support they can receive.” (I07)*

Additionally, some participants asserted that many refugees and asylum-seekers are unaware of their fundamental rights to healthcare. This, coupled with the lack of legal status in Malaysia, has undermined their ability to voice or seek formal recourse for any problems they may face throughout the healthcare seeking experience. As a result of poor awareness, the lack of health literacy and protection of one’s rights, refugees and asylum-seekers can become particularly vulnerable to further health risks.

*“I think in terms of what are the challenges [...] it’s also because of the education [...] among the refugee population. Sometimes, the refugee may not know they actually have the right to access the health facility, so they don’t seek help [...] it is also due to lack of information provided.” (I07)*

Even when a refugee or asylum-seeker patient is able to reach and receive services, some participants suggested that they may not fully understand the health information relayed to them or the treatment options available. This lack of understanding can be detrimental to their overall health seeking experience and also lead to ethical concerns in the delivery of services. In one example, a participant iterated that refugee patients often provide their signatures on consent documents for medical procedures and treatment that they may not fully understand due to language barriers.

*“There’s a lot of problem with language as well [...] most of the time, even if they signed some document, they do not know what they are signing […] without a proper interpretation or translation available.” (I01)*

As a result of poor health literacy and language barriers, refugees and asylum-seekers are highly dependent on their community leaders or members as information sources on how to navigate the health system and as translators when seeking medical care at public health facilities. As such, to address issues such as poor health literacy, one participant highlighted the importance of engaging community leaders from refugee populations of different ethnicities to identify the challenges faced by the community, in particular among the new arrivals. Additionally, refugee clinics run by NGOs are perceived to play an important role in facilitating the navigation process through its clinical referrals that are accompanied with information on where and how to seek further healthcare services at government public health facilities. When necessary, they are also accompanied by a refugee medical coordinator from the NGO clinic to assist with translation. However, only a small proportion of refugees and asylum-seekers access services at NGO clinics. As described by one participant, refugees and asylum-seekers may lack awareness on the availability of these NGO clinics:

*“I think not many refugees know about the clinic, because the figures given by the UNHCR are actually quite a lot of refugees, but [those that] come to the clinic […] monthly, around 1,000 plus, 1,200 to 1,500. So where’s the other patients? Most of the new cases, when I asked them, 'Why didn't you come here to see?' 90% said they didn't know the clinic exists.” (I05)*

### ‘Acceptability and Ability to Seek’: Language difficulties and cultural differences as barriers to access

This theme relates to the social and cultural factors that influence the refugee patient’s ability to seek and accept healthcare services [1]. Language, as a socio-cultural factor, is a key determinant in influencing the overall healthcare experience for refugees and asylum-seekers, as described by many participants. Language difficulties were perceived as a significant barrier in the health seeking process, leading to communication difficulties and misunderstandings with local healthcare staff during medical and administrative procedures. For example, medical history taking is particularly challenging with a refugee or asylum-seeker patient who cannot speak and understand the local language.

Apart from language constraints, some participants iterated that the public health services are rarely tailored to consider the cultural beliefs and traditional practices of the refugee and asylum-seeker population. For instance, many refugees and asylum-seekers are accustomed to seeking alternative treatment such as traditional medicines based on their ethnic and cultural practices, but these alternative forms of treatment are not available or offered by existing service providers.

*“Some services, the need itself is great within the refugee community but it is not available in the host setting. For example, like some traditional medicines.” (I07)*

In relation to the differences in socio-cultural background, some participants shared that refugees and asylum-seekers may at times face prejudices against them in healthcare settings due to their nationality.

*“Sadly speaking, […] I do have patients or refugees in my clinic telling me that, ‘No, they don’t treat me as a human being sometimes because of my look, because of my birth place.’” (I01)*

### ‘Availability and Ability to Reach’: Protection barriers to access; and poor healthcare access in immigration detention centers

This theme is associated with the existence of healthcare services and whether these services can be reached by the refugee and asylum-seeker population [1]. In general, most participants asserted that public healthcare facilities and services in Malaysia are widely available, especially in urban settings where most refugees and asylum-seekers reside. Malaysia was described as a country with one of the highest number of public and private healthcare facilities. For this reason, participants mostly felt that the Malaysian health system possesses the capacity to meet the health demands of the approximately 150,000 refugees and asylum-seekers, who are only a small fraction of the 31 million population living in Malaysia. In fact, some participants also pointed out that despite the legal circumstances for refugees and asylum-seekers in Malaysia, this population is provided reasonable healthcare treatment in the country.

However, physical barriers do exist for refugees and asylum-seekers in the process of reaching healthcare services. Overall, many participants expressed concerns on the protection issues that refugees and asylum-seekers face when seeking healthcare. Asylum-seekers, in particular those who have not obtained registration with UNHCR, are often fearful of seeking treatment due to a lack of documentation as this puts them at heightened risk of arrest and detention. They may be afraid of encountering authorities or raids during their travel to the healthcare facility or fear arrest if they are unable to pay the medical fees. Some participants also expressed unease over the set-up of immigration counters at some public hospitals for the purpose of facilitating the arrests of undocumented migrants after they are treated. Participants highlighted that this has further deterred refugees and asylum-seekers from seeking healthcare, even in cases of emergencies. Due to the overall precarious protection environment, many participants stressed that it is critical for refugees and asylum-seekers to possess a UNHCR card in Malaysia, as this significantly mitigates the protection risks and fear of seeking healthcare.

*“It depends also on which hospital. […] they've got the immigration counter there, so automatically, for those with no ID, they will report to [immigration].” (I05)*

Some participants also reported on the availability of static and mobile clinics run by NGOs. While these facilities may be limited in the number of clinics available, they were considered easy to reach given that most are located at areas where refugee and asylum-seeker communities reside. However, one participant shared that some of these mobile clinics were established nearby public healthcare facilities and thus, were rarely accessed by the refugee population. This participant perceived that this reflects the lack of understanding among the civil society on the existing healthcare system, as well as a lack of integration between health services provided by NGOs and the MoH. Nonetheless, some participants emphasized the need for NGO clinics as protection barriers at point of care is mitigated in such settings. Additionally, the cost of medical care is very affordable or free in some clinics, which is an important consideration for the refugee and asylum-seeker population.

*“Refugee actually is limited in their ability to seek treatment in the government hospital; outside is very expensive, so here [NGO clinic] is totally free.” (I05)*

Some participants also spoke about the lack of access and availability of healthcare services for refugees and asylum-seekers detained in immigration detention centers (IDCs). Most participants described the conditions in the IDCs as extremely dire due to poor sanitation and hygiene conditions, the lack of proper food and clean drinking water, and the constant overcrowding of detainees. Apart from the negative implications of this unconducive environment on the health of refugees and asylum-seekers who are in IDCs, participants also raised concerns on the spread of infectious diseases. According to a few participants, the common morbidities in IDCs include skin infections, respiratory tract infections, TB, infectious diarrhea, leptospirosis, physical injuries from fights, and complaints of headaches due to poor ventilation. Although health problems are widespread in IDCs, access to healthcare services are limited due to the lack of human resources to provide onsite medical care. It was also reported that detainees may encounter long waiting periods to see a doctor or be sent to the hospital for further investigation or treatment. Nonetheless, a medical assistant is usually stationed at most IDCs who can perform basic clinical tasks including first aid, blood pressure and glucose measurements, wound dressings and the prescription of some medications. Participants asserted that the medical assistant plays a key role in providing basic and emergency care in IDCs, and in making recommendations on further referrals to the hospital.

### ‘Affordability and Ability to Pay’: Financial difficulties as a key barrier to access

This theme reflects the economic capacity of the refugee and asylum-seeker population in affording the cost of healthcare services [1]. Overall, most participants highlighted that the inability to afford the costs of healthcare is a key barrier for refugees and asylum-seekers in accessing healthcare services. Participants asserted that financial difficulties in affording healthcare are exacerbated by a lack of livelihood, healthcare insurance, and/or refugee status which qualifies them for the discounted rate at public healthcare facilities. These determinants along with the exorbitant foreigner fees and requirements to pay hefty deposits prior to hospital admissions all serve as significant barriers to healthcare seeking for refugees and asylum-seekers in Malaysia.

*“The main problem [for access] - the patient even tell me - is money. They don't have money […] They can't earn a living; they have to live on - I don't know what they live on, actually […]. So they don't have money basically, and to sometimes do tests, I have to ask them, negotiate with them; 'What tests you want - you are able to do?' So we've got a lot of limitations […] even follow-up is also a bit difficult; we have to cater to their budget.” (I10)*

Participants also mentioned that the issue of affordability has worsened considerably for refugees and asylum-seekers over the recent years with the increase in medical fees for foreigners. Additionally, refugees and asylum-seekers may face difficulties with affording the transportation costs for travelling to healthcare facilities, especially if they suffer from chronic conditions such as TB or end-stage renal disease which requires follow-up several times a week for ‘directly observed therapy’ (DOT) treatment or dialysis. It was also reported by a few participants that in IDC settings, detainees are required to pay if they intend to see a doctor which inhibits their ability to access healthcare services while detained. Additionally, some participants iterated that healthcare seeking may not be a main priority for refugees and asylum-seekers as many of them are struggling to survive and the little that they earn is used to meet other basic needs such as food and shelter for themselves and their families.

*“I think they’re just striving to survive and surviving means basic needs – physical, shelter, food, get a job. I don’t think they even think of [health] – maybe they do think of health when they fall sick.” (I08)*

In regard to one’s ability to pay, many participants also spoke about the potential of health insurance initiatives such as REMEDI in improving access to healthcare by mitigating the financial burden of affording healthcare costs. Nonetheless, participants acknowledged that the healthcare coverage under REMEDI is limited and challenges remain in convincing refugees and asylum-seekers to enroll.

*“The insurance apparently is only for inpatients; you have to stay in. It's not for outpatients. […] we have this cancer lady who has insurance, but the insurance is only for 10,000, which is really not enough. […] So she had to go and find her own money and beg and borrow.” (I06)*

### ‘Appropriateness and Ability to Engage’: Need for access beyond basic essential services; and lack of engagement due to communication difficulties

This theme relates to the experience of the healthcare seeking process once the refugee or asylum-seeker is able to access services, denoting issues of quality and the nature of interpersonal relationships between healthcare provider and patient [1]. Although healthcare services were considered largely available to refugees and asylum-seekers, some participants argued that a broader perspective is needed. Beyond the availability of basic healthcare services including emergency and acute healthcare, primary healthcare and childbirth; a few participants emphasized that the provision of comprehensive healthcare needs for the population is still lacking. This was a key concern among participants as a significant proportion of the refugee and asylum-seeker population reside in Malaysia for a protracted period while awaiting registration with UNHCR or resettlement in a third country. Further, many refugees are faced with a lack of durable solutions for their situation and continue to live in Malaysia for extended periods of time. According to participants, addressing the comprehensive health needs requires a long-term approach to ensure refugees and asylum-seekers have access to various services throughout the continuum of care including antenatal care, preventive care and health education, family planning, rehabilitative and palliative care, mental health and psychosocial support etc.

*“[…] comprehensive health needs are still existent, being that the population is going to be here for a protracted period of time, we can't just focus on just access to just basic services; we also need to look at the more comprehensive needs for the long-term, which will then include all the other areas.” (I09)*

As described by some participants, the overall quality of healthcare in Malaysia is satisfactory and refugees are given reasonable healthcare treatment despite the overall legal environment. Nonetheless, participants reported that the health seeking experiences of refugee and asylum-seeker patients are hindered by multiple barriers. As mentioned above, language difficulties are a key challenge in the overall healthcare seeking experience for refugees. Many participants indicated that these language barriers lead to poor communication and a lack of information sharing between healthcare provider and patient in both administrative and treatment processes. As a result, refugee patients may not feel included or engaged in the overall healthcare seeking experience.

### Negative implications of poor healthcare access

Many participants also spoke about the various implications of poor healthcare access among refugees and asylum-seekers. One of the key issues raised was that most refugee and asylum-seeker patients only seek treatment at a very late stage, which leads to significantly poorer health and treatment outcomes. Other concerns were associated with the increase in home deliveries, the spread of infectious diseases including TB, poorer control of non-communicable diseases leading to secondary complications, loss to follow-up and poorer adherence rates among chronic care patients, and poorer quality of life and psychological health. For example, one participant described that due to access barriers and poor health literacy, it is not uncommon that pregnant mothers with HIV only approach health facilities in their third trimester for antiretroviral treatment which significantly increases the risk of mother-to-child transmission of HIV.

*“I think we've not seen the heights of the consequences of this yet, because we don't know how many more TB cases may not be getting access to care […] It will eventually encourage people to have home deliveries, which is not a great thing […] it's a little bit worrisome.” (I09)*

*“Previously, it was just coming to collect their [HIV] medication, but now it's also coming for follow-ups; even sometimes they might miss, because they don't have the money. So that's quite scary.” (I10)*

Overall, most participants expressed that the implications of poor healthcare access among refugees and asylum-seekers can be significant. Not only does it lead to poorer health outcomes including increased morbidity and mortality rates among refugee and asylum-seeker population; efforts in communicable disease control and prevention among the host population can also be hampered. Participants also perceived that the MoH’s cost-cutting measures targeting the foreigner fees and the lack of focus on comprehensive healthcare for refugees and asylum-seekers would only lead to greater financial costs to the health system in the long run.

## Discussion

This qualitative study addresses a gap in existing literature by identifying the key health concerns and barriers to healthcare access among the refugee and asylum-seeker population in Malaysia through the lens of healthcare professionals, program managers or executives, and experts on refugee and migrant health. Overall, participant narratives highlight the complex health needs of refugees and asylum-seekers with health concerns ranging from communicable to non-communicable diseases, malnutrition, maternal and child health related conditions and psychosocial disorders. Results from a health access and utilization survey conducted by UNHCR Malaysia in 2015 reported that 23.9% of the adult refugee population had hypertension and 8% had diabetes [20], while another review presented that 7% of the population had cardiovascular diseases [12]. Additionally, our findings identified mental health conditions as a concern among refugees and asylum-seekers. As described in a qualitative study, refugees in Malaysia experience significant psychological turmoil and negative mental health outcomes due to the hardship, trauma, discrimination, uncertainties of their future and socio-economic circumstances they are faced with [10]. At present, there are no studies reporting on the prevalence of communicable diseases such as TB and HIV among refugees and asylum-seekers in Malaysia, although it is well-established in the literature that Malaysia is a country with an intermediate TB burden [21]. Apart from these health problems, our study identified malnutrition among children as a key concern. Similarly, a study exploring the nutritional status of Rohingya refugee children in Malaysia reported that 27.5% were underweight and 11.5% stunted [7]. On the whole, the findings of our study add to existing literature on the multiple and complex health concerns of refugees and asylum-seekers, which highlights the need for greater attention.

Our findings reveal that multiple factors are at play in affecting the health status of refugees and asylum-seekers living in Malaysia. Apart from the health risks associated with the pre-departure and travel phases of the migration process, limited access to comprehensive healthcare post-arrival remain a fundamental problem for refugees and asylum-seekers in Malaysia. A systematic review examining non-communicable diseases among urban refugees in developing countries reported that despite good access to primary healthcare, access to secondary and tertiary healthcare is problematic [12]. This is consistent with our findings in Malaysia that accentuates the need to move beyond the provision of emergency and basic healthcare to ensure that the comprehensive health needs of refugees along the care continuum are also addressed. Furthermore, a number of studies have highlighted that a significant proportion of refugees in Malaysia are facing protracted displacement [11, 22]. In addition to the risk factors associated with urbanization among refugee communities that result in the spread of communicable diseases, population ageing due to the protracted situation also leads to the emergence of non-communicable diseases resulting in a double burden of disease [13]. This justifies the imperative for a long-term and comprehensive approach in addressing the health needs of refugees and asylum-seekers, as established from our findings.

In the overall context of healthcare access, multiple barriers still exist for refugees and asylum-seekers. Our findings indicate that poor health literacy and the lack of awareness on one’s right to healthcare are impediments to navigating the health system effectively. Many studies have shown that low health literacy is inextricably linked to poorer health outcomes [23], which is a common concern among refugee and asylum-seeker populations [24–26]. Despite such notions, UNHCR’s healthcare access and utilization survey in 2015 reported that 79.1% of refugee and asylum-seeker households were aware of the subsidized access for refugees to governmental health services [20]. Nonetheless, this finding may not provide a comprehensive depiction of the level of health literacy among refugees and asylum-seekers in Malaysia, as the lack of awareness may be more predominant among new arrivals or those lacking community support. Our findings also emphasized language differences, acculturation difficulties as barriers to the access and acceptability of healthcare services. Other studies corroborate these findings [10–12, 20, 27], thus highlighting the key importance of addressing the various social and cultural determinants of health when tackling access issues among the refugee and asylum-seeker population. Additionally, despite the good availability of public health facilities, protection concerns due to a lack of legal status remains a key obstacle for refugees and asylum-seekers in seeking and reaching healthcare services. As refugees and asylum-seekers do not possess legal identity in Malaysia, they often feel like outcasts in the society and live in constant fear and anxiety of arrest and detention [10]. Among the various barriers, our study identified financial difficulties as among the most significant, particularly for those with chronic conditions that require regular follow-up. This was accentuated in the 2015 survey conducted by UNHCR Malaysia, whereby only 70.6% of those seeking healthcare services for chronic illnesses were able to afford treatment fees, and 52.9% faced difficulties affording transportation fees to the health facility [20]. Nonetheless, existing efforts in offering healthcare insurance such as REMEDI to refugees and asylum-seekers has shown potential in reducing the financial barriers to access, although the coverage is limited, and uptake is low.

Overall, the access barriers presented in this study have numerous negative public health and social implications, particularly for the refugee and asylum-seeker population. Apart from poorer health outcomes, our findings support existing postulations that inadequate healthcare access interacts with the lack of social rights, thus reinforcing exclusion as a primary cause to poor health among refugee populations [2]. On the contrary, there can be substantial benefits when refugees and asylum-seekers are able to access affordable healthcare as needed. In a study exploring HIV outcomes among refugees in Malaysia; providing highly active antiretroviral therapy (HAART) to refugee populations on an equitable basis was proven to be both feasible and beneficial in sustaining good treatment outcomes [27]. Evidently, the public health benefits of communicable disease control extend also to the host population. Apart from restricted access to healthcare, our findings also suggest that cost-cutting measures may lead to a greater financial cost to the health system in the long term. Studies worldwide have presented some evidence for this finding [28–30]. For instance, a quasi-experimental study in Germany on the effect of limiting access to healthcare for asylum-seekers and refugees identified that the excess expenditure attributable to the exclusion of the population from healthcare was ultimately higher than granting regular access to care [28]. On the whole, our findings add to the existing literature that poor healthcare access among refugee and asylum-seekers can lead to adverse implications including threats to public health and increased healthcare costs.

### Strengths and limitations

To our knowledge, this is the first study to investigate the health concerns and barriers to access among refugees and asylum-seekers in Malaysia. The findings of this study provide novel and useful insights which has many practical implications for informing policy and interventions directed at addressing the health needs of the refugee and asylum-seeker population. Another strength of this study is in the data collection process of interviewing and capturing the viewpoints of a wide range of actors from different sectors and agencies involved in refugee and migrant health. Additionally, the use of semi-structured in-depth interviews allowed for a comprehensive exploration of important issues that emerged beyond the scope of the interview guide.

However, given the aim of this study which explores issues on healthcare access, it would be ideal to conduct interviews with refugees and asylum-seekers to better understand the healthcare seeking experiences from their perspective. In this study, the researchers’ focus on healthcare professionals, program staff and refugee health experts as participants was partly to identify the feasibility and appropriateness of interviewing refugees and asylum-seekers in future research. The researchers have established that such research would add value to the literature and in corroborating the findings of this study. Nonetheless, interviewing healthcare professionals, program staff and refugee health experts has provided a unique perspective on the topic, teasing out broader issues relating to the gaps in the system and service delivery. Apart from this, the sample of participants for this study were individuals with a keen interest on the topic area and who were very outspoken in sharing their views. Collectively, this may have limited the breadth of perspectives and resulted in potential biases whereby participants recruited were largely sympathetic to the refugee cause. Nevertheless, it was observed that most participants maintained objectivity in their views by providing a holistic presentation of the issues discussed.

### Implications for policy and practice

This study brings to light the multifaceted healthcare needs of refugees and asylum-seekers in Malaysia and the barriers to access which demands special considerations by those responding to these needs. Firstly, greater efforts should be devoted to improving the health literacy and awareness on the right to healthcare as part of existing health promotion activities among refugees and asylum-seekers. In particular, newly arrived asylum-seekers could benefit from such initiatives especially through their community members, who are the main information source for healthcare seeking. Health education could be delivered through technology-mediated interventions to attain a more effective and broader reach. Secondly, language and communication barriers could be significantly reduced with the assistance of interpreters at healthcare facilities to aid communication between providers and patients. To bridge the differences in culture between the refugee and host population, educational initiatives focusing on cultural orientation and improved awareness on the plight of refugees and asylum-seekers among healthcare providers and the public could foster greater inclusivity. Healthcare services that are tailored to the culture and traditions of refugees and asylum-seekers including alternative treatment is still a gap that could be filled by existing service providers, particularly those with targeted services for refugees and asylum-seekers.

Thirdly, overcoming the protection barriers to healthcare access is a key challenge that will require paradigm shifts in the overall policy environment to uphold healthcare as a basic human right regardless of one’s legal status. On this basis, greater attention should also be accorded to the provision of healthcare services for those detained in immigration detention centers to facilitate better access to healthcare. Fourthly, implementing inclusive health policies at the national level that ensure services throughout the care continuum are affordable to refugees and asylum-seekers could yield benefits from a cost-effective perspective. Further research in this area is warranted given the potential economic benefit of such policies to the host country. Similarly, healthcare insurance for refugees and asylum-seekers in Malaysia such as REMEDI has shown potential in reducing the financial barriers to access and should be expanded further but with further examination on how to overcome the challenges in coverage and uptake. Finally, providing comprehensive health services beyond basic essential services entails further efforts in ensuring refugees and asylum-seekers have access at the different levels of care within the health system and along the care continuum. All in all, addressing the various social determinants of health at the policy level – such as allowing access to other basic services including legal employment and formal education – will reduce healthcare barriers substantially and foster overall improvements in the health of refugees and asylum-seekers in Malaysia.

## Conclusion

Refugees and asylum-seekers are a particularly vulnerable and marginalized group in Malaysia. Despite the availability of healthcare facilities and services in the country, inadequate access to healthcare among refugees and asylum-seekers remain a significant problem. Our study highlights that the key barriers to healthcare access are linked to poor health literacy; language and cultural differences; legal and protection issues; and the inability to afford healthcare. Evidently, these barriers lead to limited access, which has significant implications on the refugee and asylum-seeker population, its host community, the service providers and the health system at large. Overall, the evidence yielded from this study suggests that comprehensive efforts in practice and research that tackle the overall social, cultural and economic determinants of health for more inclusive policies is crucial in strengthening healthcare access among refugees and asylum-seekers in Malaysia.

## Abbreviations

DOT: Directly observed therapy
HAART: Highly active antiretroviral therapy
IDC: Immigration detention center
MoH: Ministry of Health
NGOs: Non-governmental organizations
NUS-IRB: National University of Singapore Institutional Review Board
UNHCR: United Nations High Commissioner for Refugees

## References

[1] Levesque J-F, Harris MF, Russell G (2013). Patient-centred access to health care: conceptualising access at the interface of health systems and populations. International Journal Equity Health.

[2] WHO Regional Office for Europe. How health systems can address health inequities linked to migration and ethnicity. . In*.* Copenhagen: WHO; 2010.

[3] Zimmerman C, Kiss L, Hossain M (2011). Migration and Health: A Framework for 21st Century Policy-Making. PLoS Med.

[4] Shum K, UNHCR (2016). Regional Office for South-East Asia. Mixed maritime movements in South-East Asia in 2015.

[5] Wake C (2014). Forced migration, urbanization and health: exploring social determinants of health among refugee women in Malaysia.

[6] Nah A (2010). Refugees and space in urban areas in Malaysia. Forced Migration Review.

[7] Teng TS (2011). Nutritional status of Rohingya children in Kuala Lumpur. Malaysian Journal of Medicine and Health Sciences.

[8] Hoffstaedster G (2014). Place-making: Chin refugees, citizenship and the state in Malaysia. Citizenship Studies.

[9] Letchamanan H (2013). Myanmar’s Rohingya Refugees in Malaysia: Education and the Way Forward. Journal of International and Comparative Education.

[10] Low SK, Kok JK, Lee WY (2014). Perceived Discrimination and Psychological Distress of Myanmar Refugees in Malaysia. International Journal of Social Science and Humanity.

[11] Wake C, Cheung T (2016). Livelihood strategies of Rohingya refugees in Malaysia ‘we want to live in dignity’.

[12] Amara AH, Aljunid SM. Noncommunicable diseases among urban refugees and asylum-seekers in developing countries: a neglected health care need. Glob Health. 2014:10(24). 10.1186/1744-8603-10-24 PMC3978000 24708876

[13] Spiegel PB, Checchi F, Colombo S, Paik E (2010). Health-care needs of people affected by conflict: future trends and changing frameworks. Lancet.

[14] The UN Refugee Agency. Malaysia: UNHCR global appeal 2012-2013. In: The UN Refugee Agency; 2013.

[15] UNHCR. Figures at a Glance in Malaysia [http://www.unhcr.org/en-my/figures-at-a-glance-in-malaysia.html]. Accessed: 02 July 2018.

[16] Brinham N, Chickera AD, Petrova D, The Equal Rights Trust (2014). Equal only in name: the human rights of stateless Rohingya in Malaysia.

[17] International Federation of Red Cross Red Crescent Societies. REMEDI - refugee medical insurance, Malaysia [http://media.ifrc.org/global-review-on-migration/smart-practice/remedi-refugee-medical-insurance-malaysia/]. Accessed: 15 June 2018.

[18] Hamdan N. Policy to address medical issues faced by migrants in Malaysia, says UNHCR rep. In: The Star Online. Kuala Lumpur; 2016.

[19] Puras D (2015). Report of the special rapporteur on the right of everyone to the enjoyment of the highest attainable standard of physical and mental health. Human rights council, twenty-ninth session.

[20] UNHCR Malaysia (2015). At a glance: health access and utilization survey among non-camp refugees in Malaysia.

[21] World Health Organization (2013). Tubercolosis country profiles.

[22] Azis A (2014). Urban refugees in a graduated sovereignty: the experiences of the stateless Rohingya in the Klang Valley. Cuitizenship Studies.

[23] Berkman ND, Sheridan SL, Donahue KE, Halpern DJ, Crotty K (2011). Low health literacy and health outcomes: an updated systematic review. Ann Intern Med.

[24] Wångdahl J, Lytsy P, Mårtensson L, Westerling R (2015). Health literacy and refugees’ experiences of the health examination for asylum seekers – a Swedish cross-sectional study. BMC Public Health.

[25] Riggs E, Yelland J, Duell-Piening P, Brown SJ (2016). Improving health literacy in refugee populations. Medical Journal Australia.

[26] Sheikh-Mohammed M, MacIntyre CR, Wood NJ, Leask J, Isaacs D (2006). Barriers to access to health care for newly resettled sub-Saharan refugees in Australia. Medical Journal Australia.

[27] Mendelsohn JB, Schilperoord M, Spiegel P, Balasundaram S, Radhakrishnan A, Lee CKC, Larke N, Grant AD, Sondorp E, Ross DA (2014). Is forced migration a barrier to treatment success? Similar HIV treatment outcomes among refugees and a surrounding host Community in Kuala Lumpur, Malaysia. AIDS Behavior.

[28] Bozorgmehr K, Razum O (2015). Effect of Restricting Access to Health Care on Health Expenditures among Asylum-Seekers and Refugees: A Quasi-Experimental Study in Germany, 1994–2013. PLoS One.

[29] FRA European Union Agency for Fundamental Rights (2015). Cost of exclusion from healthcare: The case of migrants in an irregular situation.

[30] Britz JB, McKee M (2016). Charging migrants for health care could compromise public health and increase costs for the NHS. Journal of Public Health.

